# Molecular epidemiology and characterization of goose polyomavirus in China: insights into its impact on hatchability and susceptibility to co-infections

**DOI:** 10.3389/fvets.2026.1810451

**Published:** 2026-05-12

**Authors:** Yadong Gao, Lintao Fang, Yan Wang, Qingru Chang, Lingna Zhang, Junjie Guo, Jingyi Wu, Xinran Yao, Jiaqi Liu, Junwei Ge

**Affiliations:** 1 Heilongjiang Provincial Key Laboratory of Zoonosis, College of Veterinary Medicine, Northeast Agricultural University, Harbin, Heilongjiang, China; 2 College of Veterinary Medicine, Gansu Agricultural University, Lanzhou, China; 3 School of Life Sciences, Jilin University, Changchun, China

**Keywords:** co-infection, goose hemorrhagic polyomavirus, molecular epidemiology, outcome, whole genome sequencing

## Abstract

**Introduction:**

Goose hemorrhagic polyomavirus (GHPV) is the primary etiological agent of goose hemorrhagic nephritis and enteritis (HGNE), a disease causing substantial economic losses to the global waterfowl industry. Despite documented outbreaks in Europe and Asia, integrated molecular epidemiological evidence linking clinical outbreaks, co-infections, and viral phylogeny remains limited.

**Methods:**

We conducted nationwide epidemiological surveillance across eight provinces in China from March 2023 to April 2025, covering 83 farms and 1,902 samples. An outbreak of reduced hatchability was investigated via PCR screening for nine common waterfowl pathogens and bacteriological culture. Virus isolation was performed using embryos from multiple goose breeds, followed by full-genome sequencing and comparative molecular characterization of the isolates.

**Results:**

GHPV was detected in 49% (41/83) of surveyed farms, with an overall individual prevalence of 4% (84/1902). No co-infections were observed in goslings or embryos, but a high co-infection rate of 75% (27/36) was found in GHPV-positive adult geese, with Goose circovirus (GoCV) as the most frequent co-infecting agent (93%). Experimental inoculation showed that Landes goose embryos and their derived primary cells were highly susceptible to GHPV replication, whereas Sichuan White and Sanhua Hybrid geese were not permissive, indicating breed-dependent host–virus interactions. The Chinese isolates exhibited high genomic conservation, with VP1 gene nucleotide homology ranging from 98.3% to 100% among global strains.

**Discussion:**

The combination of low overall prevalence, high co-infection rate in adults, and marked genomic conservation suggests that GHPV may act as an opportunistic pathogen, whose clinical impact is amplified through synergistic interactions with co-infecting agents, particularly GoCV. These findings highlight the need for integrated surveillance targeting co-circulating pathogens and underscore the importance of breed-specific considerations in hatchery management and disease control strategies.

## 1 Introduction

Goose hemorrhagic polyomavirus (GHPV), a member of the Polyomaviridae family, features a small, non-enveloped icosahedral capsid encapsidating a circular double-stranded DNA genome of approximately 5.2 kbp. This genome orchestrates the expression of structural proteins (VP1, VP2, and VP3), regulatory T-antigens, and an accessory ORF-X with a currently undetermined functional role (1). The VP1 gene encodes the major capsid protein of GHPV, which is highly conserved among polyomaviruses and exhibits strong specificity, making it a globally established and stable target for viral detection, genotyping, and nucleic acid-based diagnosis since the initial discovery of the virus (2–5). As the etiological driver of hemorrhagic nephritis and enteritis of geese (HNEG), GHPV triggers acute systemic pathogenesis. While primarily devastating goslings aged 3–10 weeks (2), the virus also affects adult populations, frequently manifesting as high-mortality outbreaks characterized by systemic vascular compromise, hemorrhagic enteritis, and severe renal lesions (6, 7). In Belgium, a case of persistent infection has also been reported (8).

Since its initial discovery in 1969 (9), GHPV has been documented across various European nations and has recently emerged as a significant concern within Asian waterfowl sectors (2, 10). In China, molecular and serological surveillance has suggested ducks as asymptomatic reservoirs of GHPV (11). The existence of these subclinical carriers complicates biosecurity frameworks and poses a persistent challenge to effective disease eradication and containment.

Despite advancements in GHPV research, contemporary studies remain largely confined to routine pathogen detection or localized genomic assessments (12–14).

Comprehensive investigations that bridge clinical outbreak dynamics, the broader epidemiological burden of GHPV, co-infection landscapes, and high-resolution molecular signatures are notably scarce. Consequently, the molecular epidemiology of GHPV across different regions remains unclear, and comprehensive genomic data from Chinese isolates are limited. Moreover, the specific role of GHPV in hatchability decline—a major cause of economic loss in goose hatcheries—has not been defined (4, 6).

In this study, we characterized a natural outbreak of impaired hatchability in a commercial goose flock in Yunnan Province, China. To delineate the causative factors, a diagnostic pipeline integrating PCR-based co-infection screening, viral isolation, and whole-genome sequencing was implemented. Furthermore, a nationwide molecular survey was executed to evaluate GHPV prevalence and its synergistic association with other pathogens. Phylogenetic reconstruction and codon usage bias analysis of the VP1 gene were further utilized to resolve the genetic architecture of the identified strains. Collectively, these findings will directly benefit prevention and control strategies by identifying high-risk regions and age groups, highlighting the importance of managing GHPV and its common co-infecting agents (e.g., GoCV) in commercial goose flocks, and revealing breed-informed biosecurity measures in goose hatcheries.

## 2 Materials and methods

### 2.1 Case history and clinical specimen collection

In June 2022, a commercial goose hatchery in Yunnan Province, Southwest China, reported a substantial decline in hatchability, with losses reaching approximately 30%. The clinical profile of the affected embryos was primarily defined by late-term mortality, an inability to pip the shell, and the production of debilitated, nonviable goslings. Throughout the observation period, incubation variables—specifically thermal and hygrometric settings—were maintained within standard operational ranges. Furthermore, the source breeding flock, which had been conventionally immunized against endemic waterfowl pathogens, remained clinically asymptomatic. Visceral specimens (kidney, spleen, liver, and intestine) were aseptically collected from ten freshly dead or moribund embryos for comprehensive laboratory analysis.

Subsequently, a systematic epidemiological surveillance program was implemented across major goose-producing hubs in eight provinces (Shandong, Yunnan, Sichuan, Jiangsu, Fujian, Zhejiang, Heilongjiang, and Anhui) between March 2023 and April 2025. Clinical samples (*n* = 1902) were procured from domestic geese exhibiting symptoms congruent with GHPV infection. All biological materials were cryopreserved at −20 °C pending molecular characterization. Detailed metadata for each specimen, encompassing temporal data, geographic coordinates, host age, clinical presentation, and immunological background, were rigorously documented.

### 2.2 Nucleic acid extraction and pathogen detection

Total DNA and RNA were extracted from approximately 200 mg of each tissue sample using commercially available kits according to the manufacturer’s instructions. To investigate potential co-infections, all samples were screened for common waterfowl pathogens, including Goose Parvovirus (GPV), Avian Influenza Virus (AIV), Duck Enteritis Virus (DEV), Goose Circovirus (GoCV), Goose Astrovirus (GoAstV), Fowl Adenovirus (FAdV), Newcastle Disease Virus (NDV), and Novel Duck Reovirus (NDRV). These screenings were performed using previously established PCR or RT-PCR protocols (15). The resulting PCR products were purified and further validated by Sanger sequencing.

Simultaneously, samples were subjected to bacteriological examination. Under aseptic conditions, tissue homogenates were inoculated onto 5% sheep blood agar and incubated both aerobically and anaerobically at 37 °C for 48 h (16). Bacterial isolates were identified through standard biochemical procedures and further confirmed using the Biolog Microbial ID System (GEN III, Biolog Inc., USA).

### 2.3 Virus isolation and electron microscopy

Liver and spleen tissues from GHPV-positive samples were homogenized in buffer A [10 mM Tris–HCl, 100 mM NaCl, 1 mM EDTA (pH 7.2)] at a weight-to-volume ratio of 1:5. The resulting homogenate was centrifuged at 10,000 × g for 30 min at 4 °C, and the supernatant was subsequently passed through a 0.22-μm membrane filter. The filtrate was supplemented with penicillin (10,000 IU/mL) and streptomycin (1 mg/mL) prior to inoculation. This inoculum was then injected into the allantoic cavities of 16-day-old goose embryos derived from three different breeds to evaluate viral permissiveness. Inoculated embryos were monitored daily for 10 days post-infection. Prior to harvesting, the embryos were chilled at 4 °C for 4 h.

For morphological characterization, viral particles were examined using a Hitachi HT7700 transmission electron microscope (TEM). Briefly, the samples were prepared according to standard procedures and observed at an accelerating voltage of 80 kV.

### 2.4 Genome sequencing and genetic analysis

A representative sample, successfully passaged in goose embryos, was selected for full-genome sequencing. The viral genome was amplified in overlapping fragments using five pairs of specific primers (listed in Table 1), which were synthesized by GenScript (Nanjing, China). The resulting PCR products were purified and subjected to bidirectional Sanger sequencing. To ensure sequence accuracy, the entire genomic assembly was performed in at least three independent replicates.

**Table 1:** Primers used for PCR amplification and sequence analysis.

Primers	Sequences (5′-3′)	Sense	Position	Amplicon size
GHPV-1F	TTCCAATTCACTTACACG	+	4,943–4,961 916–899	1,226
GHPV-1R	AGAAACTCATTACACCCG	−		
GHPV-2F	TGCTGGGACATCTTCTAC	+	673–691 1994–1977	1,321
GHPV-2R	GGTTTCTTGGTCTTTTTA	−		
GHPV-3F	CCACATTCATTACACAGA	+	1752–1770 3,176–3,159	1,424
GHPV-3R	CCAGAAAATACAGGAGAC	−		
GHPV-4F	TGGTGGGGGATAACTCTC	+	2,912–2,930 4,158–4,141	1,246
GHPV-4R	AAATCTTTGTTTTGGGCA	−		
GHPV-5F	ATGGAGCAGAGCAATAGC	+	3,889–3,907 5,152–5,134	1,263
GHPV-5R	TTCATCCTGACAAGGGAG	−		

Additionally, the VP1 gene of GHPV was amplified via conventional PCR from ten selected GHPV-positive specimens obtained from geographically distinct goose farms. Sequence alignment, polymorphism identification, and recombination analysis were conducted following established methodologies (16–19).

### 2.5 Phylogenetic analysis

The obtained VP1 gene sequences and the complete viral genome were aligned with reference sequences retrieved from the GenBank database. Phylogenetic trees were reconstructed using the Maximum Likelihood (ML) method implemented in MEGA VII software. To evaluate the reliability of the branching patterns, bootstrap analysis was performed with 1,000 replicates. To ensure the robustness of the ML tree topology, alternative reconstruction methods, including Maximum Parsimony (MP) and Neighbor-Joining (NJ), were also utilized for comparative verification.

## 3 Results

### 3.1 GHPV is the primary etiological agent in the outbreak

In the investigated case of reduced hatchability, GHPV was detected in 100% (10/10) of the symptomatic goose embryos via PCR. Conversely, all specimens tested negative for the other eight waterfowl pathogens, including GPV, AIV, DEV, GoCV, GoAstV, FAdV, NDV, and NDRV. Furthermore, no pathogenic bacteria were isolated from the visceral tissues under either aerobic or anaerobic culture conditions. After systematically excluding these common infectious agents and considering the consistency of incubation parameters, GHPV was identified as the sole pathogen present, with no evidence of co-infection. These results collectively demonstrate that GHPV was the primary etiological agent responsible for this specific hatchery outbreak.

### 3.2 Investigation and prevalence of GHPV in goose farms nationwide show differences in positive rates

The nationwide epidemiological investigation across eight Chinese provinces revealed that 49% (41/83) of the surveyed farms were positive for GHPV. The prevalence at the farm level varied geographically; the highest detection rates were observed in Yunnan (78%), followed by Anhui (67%), Jiangsu (60%), Sichuan (60%), Shandong (60%), and Fujian (50%). Conversely, no GHPV-positive cases were identified in Zhejiang or Heilongjiang provinces. Detailed characteristics of the positive specimens are summarized in Supplementary Table S1, with a comprehensive list of all sample results provided in Supplementary Table S2.

At the individual sample level, the overall positivity rate was 4% (84/1902). Specifically, the prevalence was 2.5% (48/1902) in goslings and goose embryos, compared to 1.9% (36/1902) in adult geese. Seasonal analysis indicated fluctuating infection rates, with the highest prevalence recorded in summer (7%, 17/354) and spring (6%, 34/558), followed by winter (4.8%, 11/226) and autumn (2.8%, 22/764).

### 3.3 Co-infections are prevalent and associated with severity

Screening for polymicrobial infections demonstrated distinct age-related patterns in GHPV-positive specimens. While no co-infections were detected in goslings or goose embryos, a high mixed infection rate of 75% (27/36) was observed in adult geese. As shown in Supplementary Table S3, among these cases, GoCV was the most prevalent co-infecting agent, followed by GoAstV, NDV, FAdV, GPV, and NDRV. Among mixed infections, triple infections were the most common, followed by dual and quadruple infections. The core combination patterns of the different infection types are detailed in Supplementary Table S4.

Within dual infections, the GHPV/GoCV combination predominated, followed by GHPV/GoAstV. In triple infections, the most frequent profile was GHPV co-infected with GoAstV and GoCV, followed by GHPV/GPV/GoCV and GHPV/GoCV/NDV. Among quadruple infections, the GHPV/FAdV/GoAstV/GoCV combination was identified in 80% of cases, while GHPV/NDRV/GoCV/GoAstV accounted for the remaining 20%.

### 3.4 GHPV strain can increase and propagate in the embryos and of Landes geese

**Figure 1:**
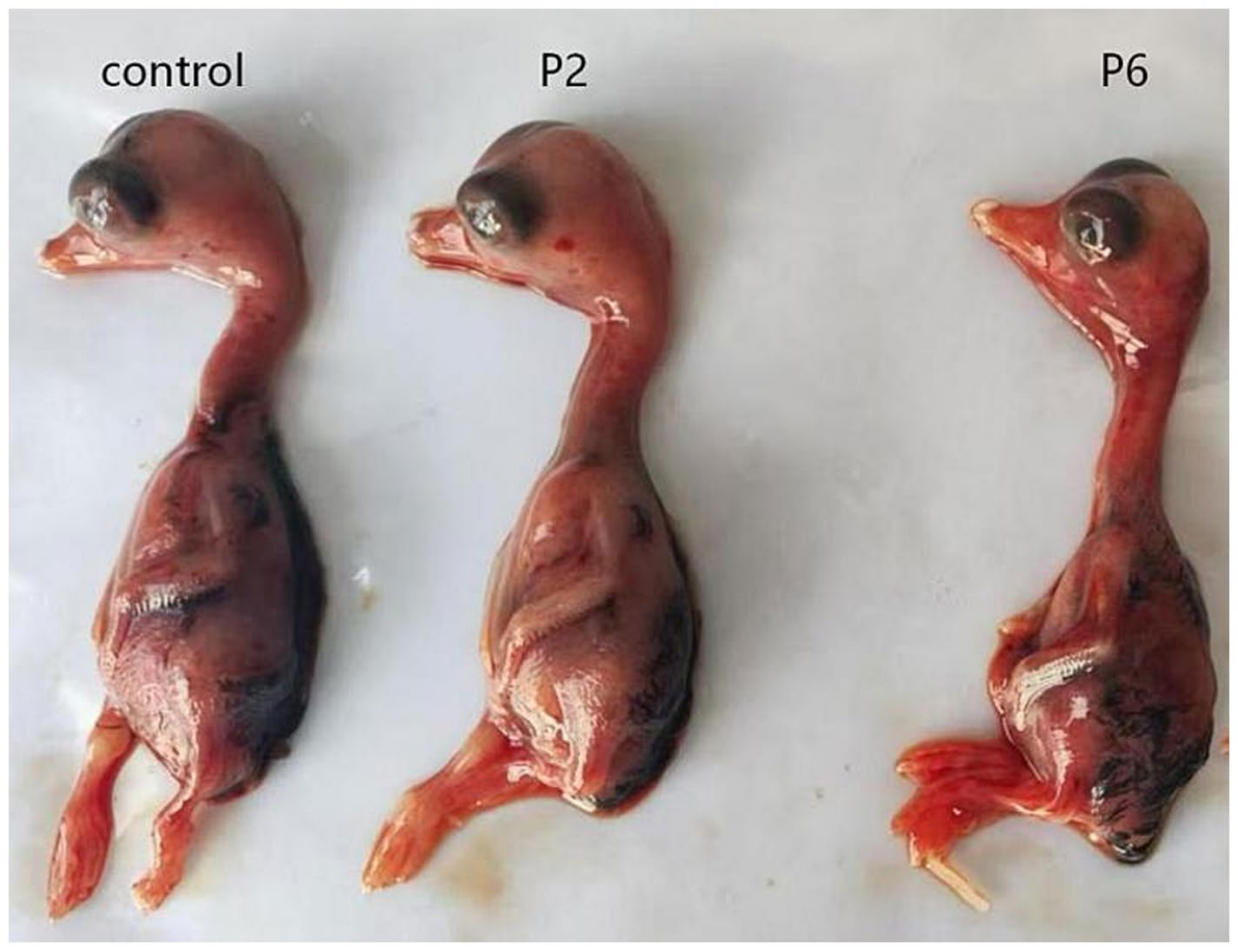
Pathological changes of Landes goose embryos infected with GHPV. The control group consisted of normal Landes goose embryos without GHPV infection; P2 and P6 represented Landes goose embryos infected with the 2nd and 6th passages of GHPV, respectively.

**Figure 2:**
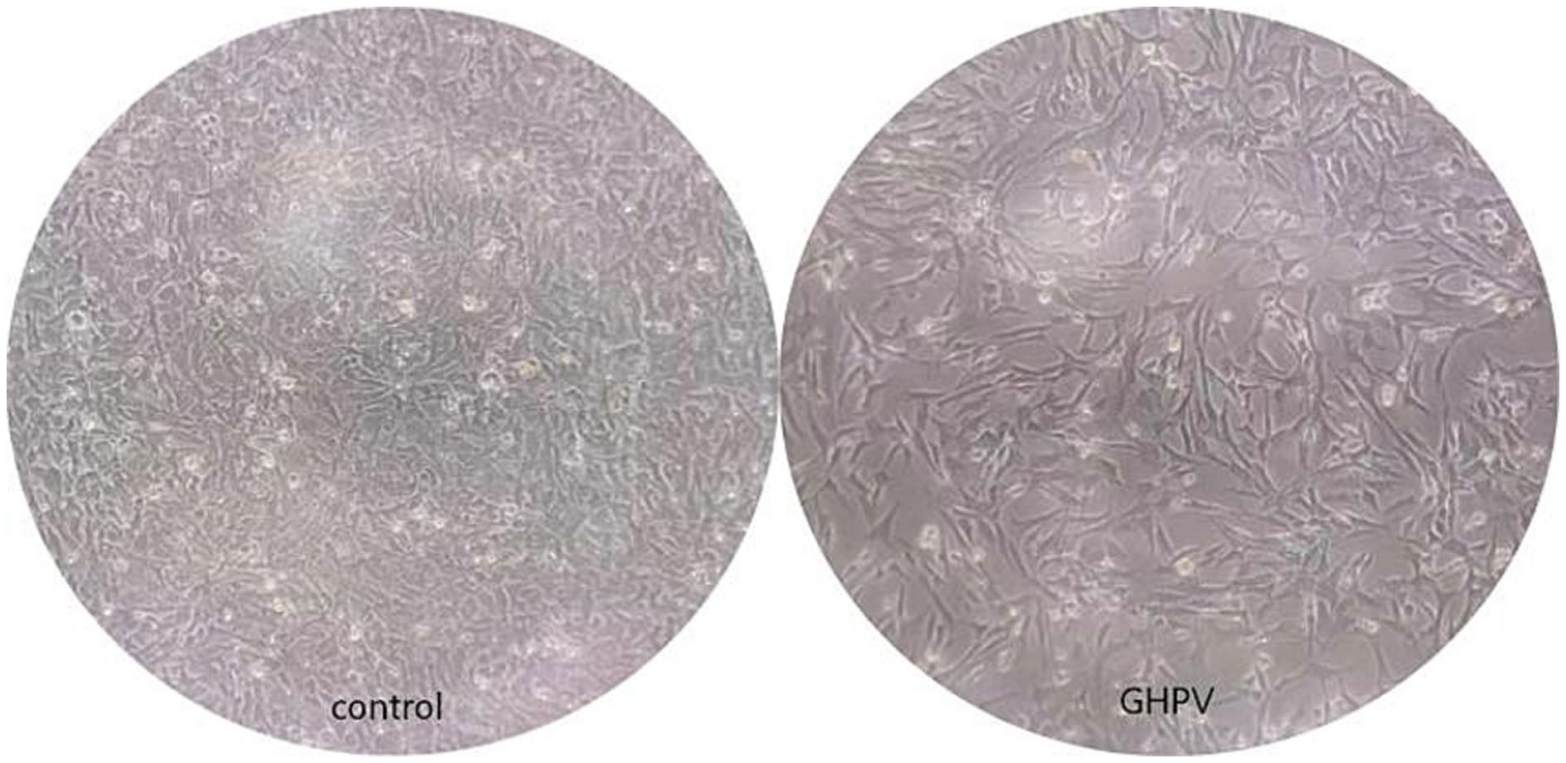
Morphological changes of Landes goose-derived primary cells infected with GHPV.

To evaluate viral susceptibility across different hosts, three breeds of goose embryos—Landes, Sichuan White, and ‌Sanhua Hybrid Goose—were inoculated for virus isolation. The results demonstrated that GHPV successfully proliferated exclusively in Landes goose embryos, whereas no viral replication was observed in the other two breeds. Inoculated Landes embryos exhibited characteristic pathological lesions, including extensive cutaneous hemorrhaging, generalized edema, and severe developmental stunting with a markedly reduced body size (Figure 1). These clinical manifestations were consistent with natural infection symptoms, and no other confounding pathogens were detected. Viral presence remained detectable even after ten consecutive passages in Landes embryos, confirming stable viral propagation. Furthermore, primary cell cultures—including goose embryo fibroblasts (GEFs), kidney cells, and liver cells—were derived from these three breeds. Consistent with the embryo inoculation results, viral isolation and cytopathic effects were only achieved in Landes-derived primary cells (Figure 2).

**Figure 3:**
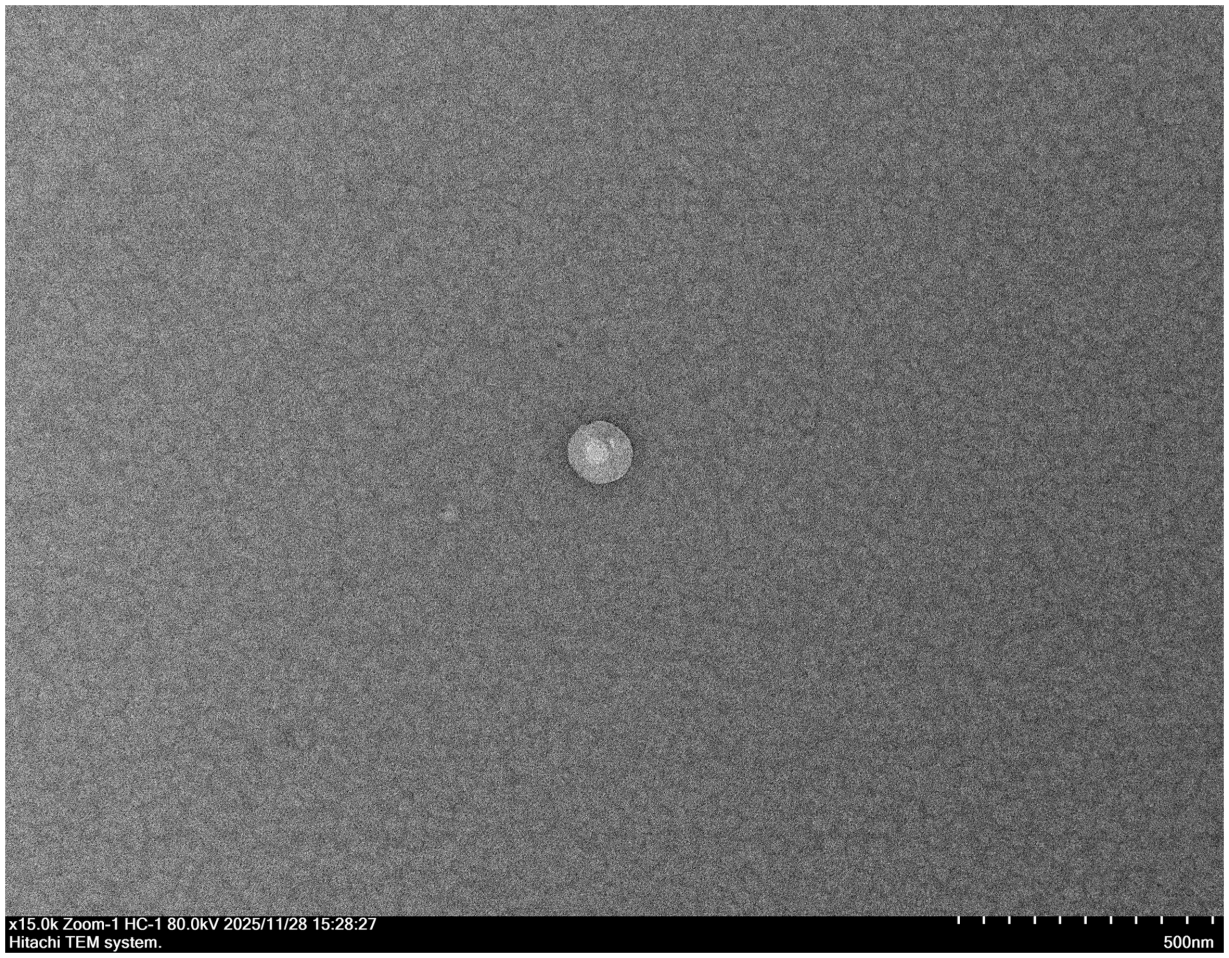
Electron microscopic examination of GHPV. Negatively stained micrograph of viral particles from goose embryo allantoic fluid. Displayed are naked virion particles 45 nm in diameter (bar = 100 nm).

Morphological analysis via negative-staining electron microscopy revealed spherical, non-enveloped virions approximately 45 nm in diameter, consistent with the structural architecture of the Polyomaviridae family (Figure 3).

### 3.5 The nucleotide and amino acid sequence alignment of VP1 shows high conservation

The complete genome of the representative isolate, GHPV/Goose/China/YN2022/2023, was characterized and deposited in GenBank (GenBank ID PX932420). The genome is 5,256 bp in length and maintains the canonical genomic organization characteristic of GHPV. Sequence identity analysis revealed that the YN2022 genome shares 99.5 to 99.8% homology with existing GHPV sequences in the database (e.g., NC_004800 and MZ614863). Comparative genomics further demonstrated that the YN2022 strain exhibits high sequence similarity with isolates from Europe (99.6–99.8%), and Asia (99.5–99.9%). These findings suggest a high degree of genomic stability across geographically diverse GHPV lineages (Supplementary Table S5).

**Figure 4:**
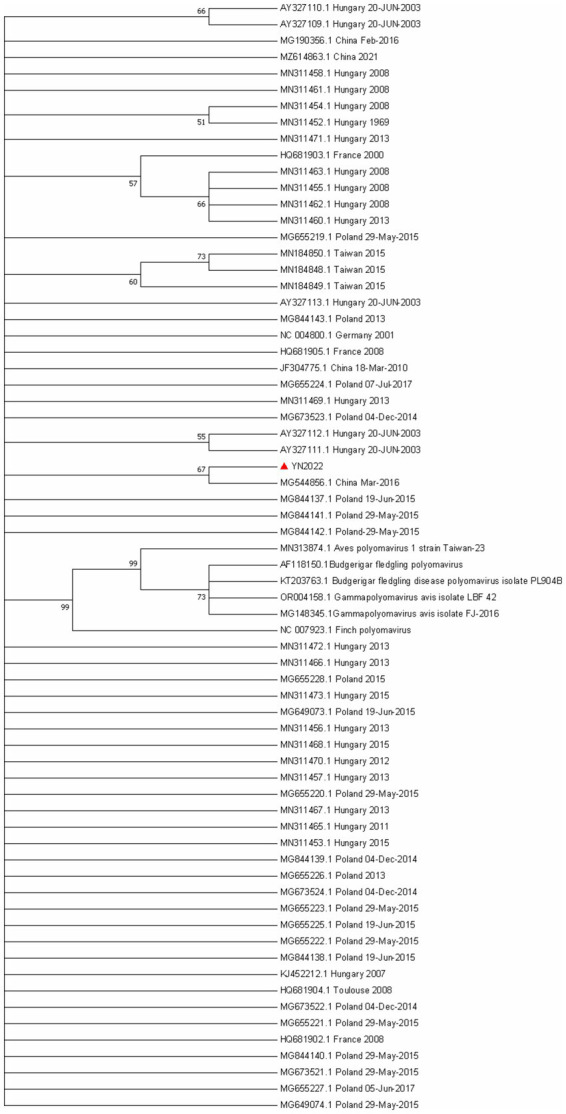
Evolutionary relationships of VP1 gene of GHPV. The phylogenetic tree was constructed using the Neighbor Joining Method in MEGA7. The percentages of replicate trees in which the associated taxa clustered together in the bootstrap test (1000 replicates) are shown next to the branches. The evolutionary distances were computed using the Maximum Composite Likelihood (MCL) algorithm, an optimized variant of maximum likelihood that enables efficient inference of evolutionary relationships from nucleotide sequence data.

To further delineate genetic variations, the nucleotide and amino acid sequences of the VP1 gene from the obtained isolates were analyzed using the MegAlign program (DNASTAR, Madison, WI, USA). A total of 62 VP1 sequences available in the GenBank were compared and found to have nucleotide homology ranging from 98.3 to 100.0% and amino acid similarity ranging from 99.5 to 100% (Supplementary Table S6). When aligned with European strains, YN2022 exhibited nucleotide homology of 98.1 to 100% and amino acid similarity of 96.9 to 100%. In comparisons with Chinese strains, nucleotide homology ranged from 99.4 to 100%, while amino acid similarity was consistently 100% (Supplementary Table S6). Additionally, the homology of ten VP1 genes obtained from geographically distinct goose farms ranges from 99.2 to 100%. Collectively, these data confirm that the VP1 gene remains highly conserved across different GHPV strains, despite subtle evolutionary mutations (Figure 4).

### 3.6 Phylogenetic analysis of GHPV

**Figure 5:**
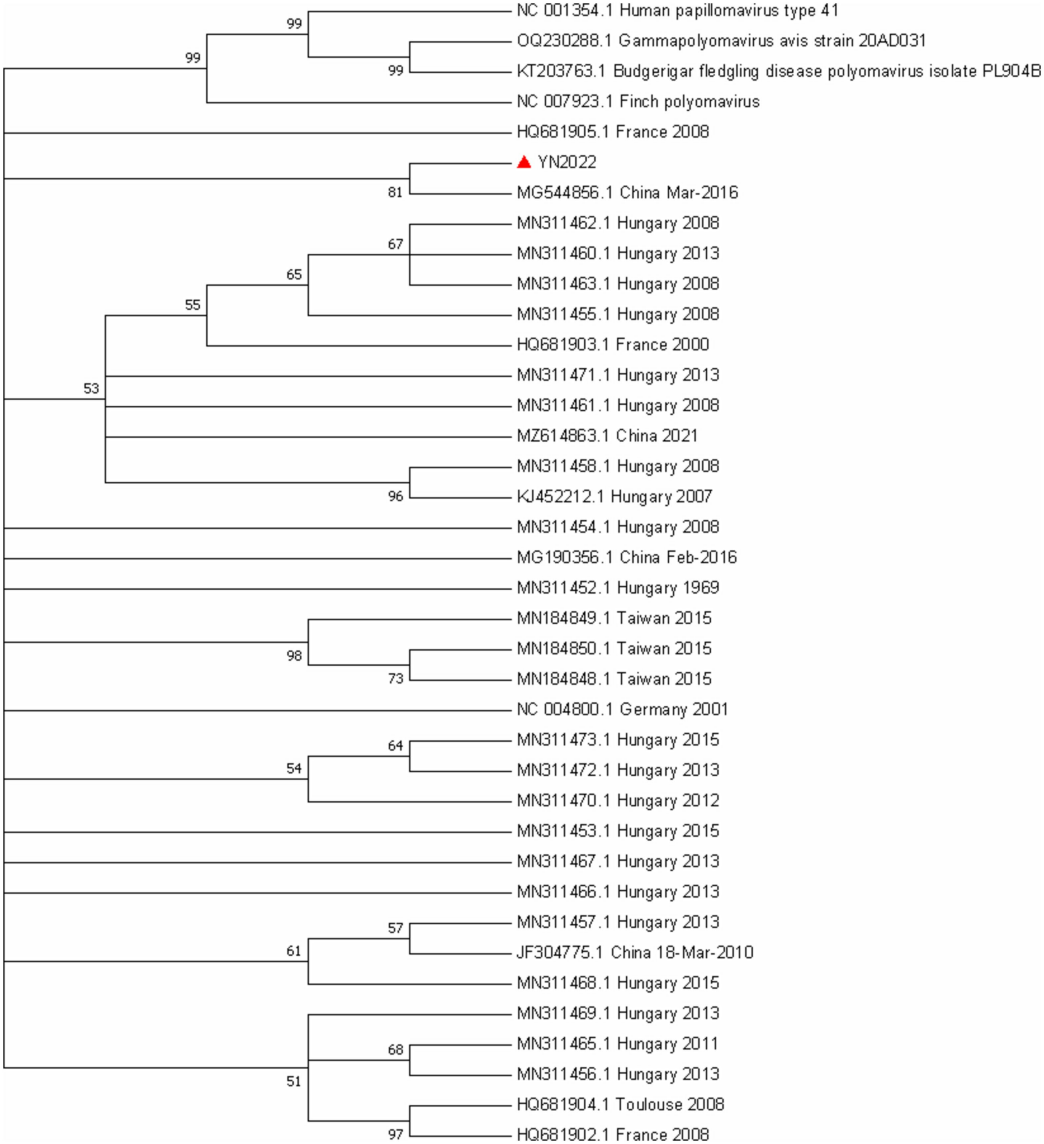
Evolutionary relationships of VP1 gene of GHPV. The plylognetic tree was constructed using the Neighbor Joining Method in MEGA7. The percentages of replicate trees in which the associated taxa clustered together in the bootstap test (100 replicates) are shown next to the branches. The evolutionary distances were computed using the Maximum Composite Likelihood (MCL) algorithm, an optimized variant of maximum likelihood that enables efficient inference of evolutionary relationships from nucleotide sequence data.

The phylogenetic relationship between the GHPV isolates and reference strains, based on complete genomic sequences, is illustrated in Figure 5. Phylogenetic reconstruction demonstrated that global GHPV strains do not cluster into distinct, time-ordered evolutionary lineages. Furthermore, isolates from disparate geographical regions exhibited no discernible temporal or spatial patterns. The YN2022 strain identified in this study showed the highest genetic proximity to the Chinese reference strain MG544856 and exhibited no significant genetic divergence from other domestic or international reference sequences (Figure 4).

No recombination events were found in the recombination analysis. To study the phylogenetic relationship, we also performed phylogenetic analysis on the basis of 68 sequences of GHPV VP1 gene. Phylogenetic analysis showed that (Figure 4) GHPV VP1 gene do not cluster into distinct evolutionary lineages, which is consistent with the high conservation of its genes.

### 3.7 Polymorphism analysis

The genetic diversity of the GHPV population was quantified using several key polymorphism indices based on 34 genomic sequences, as shown in Supplementary Table S7. The average number of nucleotide differences (*K*) was determined to be 11.148, with an overall nucleotide diversity (*π*) of 0.00212. A total of 31 distinct haplotypes (*h*) were identified within the dataset. The haplotype diversity (*Hd*) was calculated as 0.995 ± 0.009, with a variance of haplotype diversity of 0.00007.

### 3.8 Codon usage analysis

Analysis of synonymous codon usage patterns within the VP1 gene of 62 GHPV strains revealed that only AGC (Ser) and GCA (Ala) consistently exhibited Relative Synonymous Codon Usage (*RSCU*) values exceeding 2.0, indicating a distinct preference for these codons in encoding Serine and Alanine, respectively (Supplementary Table S8). In 15 specific isolates, the AGA codon was preferentially utilized for Arginine (*Arg*) with an *RSCU > 2*. Conversely, two other strains (MG655220.1 and MG844141.1) displayed divergent preferences for *Arg*, favoring the CGG and AGG codons, respectively. The Effective Number of Codons (*ENC*) for the GHPV strains ranged from 53.40 to 54.96, with a mean of 53.88 ± 0.24.

## 4 Discussion

Goose hemorrhagic polyomavirus (GHPV) is recognized as the etiological agent of hemorrhagic nephritis and enteritis in geese (HNEG) and is associated with considerable economic losses in the global waterfowl industry (20).

Despite this, current knowledge regarding the epidemiological patterns, pathogenic mechanisms, and molecular features of circulating GHPV strains remains insufficient. In the present study, an investigation was initiated following the diagnosis of a hatchery-associated case characterized by reduced hatchability, which underscored the potential reproductive impact of the virus. This initial observation prompted successful viral isolation, subsequent pathogenic characterization, and a comprehensive nationwide molecular epidemiological survey.

Previous studies have established that GHPV induces systemic hemorrhage and is lethal to goslings (2). However, its specific pathogenic role in embryonic development and hatching success remains largely undefined (7). In the investigated hatchery outbreak, GHPV was detected in 100% of symptomatic goose embryos, while all other common waterfowl pathogens, including GPV, AIV, DEV, and GoCV, tested negative, confirming that GHPV acted as the primary pathogen in early developmental stages. After a rigorous exclusion of nutritional, environmental, and bacteriological factors, GHPV was identified as the sole infectious agent in the initial hatchery case, providing strong evidence that it can act as a primary pathogen causing embryo mortality and reduced hatching rates. Our findings thus provide new evidence that GHPV can act as a primary pathogen causing embryo mortality and reduced hatching rates. These results were further corroborated by viral isolation experiments, in which GHPV passaged in Landes goose embryos induced pathological changes that closely mirrored the clinical manifestations observed in the field. Collectively, these data confirm that GHPV is a significant pathogen affecting the incubation phase in geese.

Active molecular surveillance across major goose-producing provinces revealed a relatively low overall prevalence of GHPV, yet highlighted a heterogeneous geographical distribution (21). Significant regional variations were observed, with markedly higher positivity rates in the southwestern provinces of Yunnan and Sichuan compared to other regions. In contrast, no GHPV-positive cases were identified in Heilongjiang Province in the Northeast, and no positives were detected in Zhejiang, indicating that both regional environmental factors and local biosecurity practices may influence viral circulation. Furthermore, the substantial inter-farm variability in detection rates underscores the localized nature of GHPV outbreaks. Seasonal analysis of individual samples showed fluctuating infection rates, with no consistent pattern, suggesting that GHPV persistence is largely independent of climatic fluctuations. These findings, combined with our nationwide survey showing farm-level prevalence from 0 to 78% across provinces, indicate that GHPV circulation is geographically heterogeneous, and highlight the value of targeted regional monitoring for early detection and management. Additionally, the epidemiological data revealed an age-related pattern: viral detection rates and clinical severity were higher in younger geese, particularly goslings and embryos, whereas older geese showed lower detection rates and predominantly mixed infections. This suggests that GHPV pathogenicity declines with host age, and that clinical outcomes in adult geese may be largely influenced by co-infecting agents such as GoCV. These findings highlight a significant correlation between host age, co-infection status, and clinical severity. Although GHPV detection rates appeared to decrease with age, monoinfection was predominantly associated with non-fatal cases. In contrast, mortality rates were significantly higher in geese harboring GHPV/GoCV co-infections compared to those with GHPV monoinfection, suggesting that GHPV acts synergistically with other pathogens to exacerbate disease outcomes. Clinical observations from the field further support this, noting that while certain viral infections may be manageable through supportive care, GHPV co-infection markedly increases treatment difficulty and reduces the efficacy of standard therapeutic interventions.

Field diagnostics further indicated that GHPV infection was associated with mortality in both goose embryos and young goslings, demonstrating pronounced lethality at early developmental stages. In contrast, among older geese, GHPV was detected exclusively in deceased individuals and consistently in the context of mixed infections. This age-related pattern, supported by co-infection data showing a 75% mixed infection rate in adults predominantly involving GoCV, indicates that while GHPV is highly pathogenic in early stages as a monoinfection, its clinical severity in older geese is largely influenced by synergistic interactions with immunosuppressive viruses. Moreover, clinical testing demonstrated a positive correlation between viral detection rates and the severity of clinical manifestations observed in affected flocks.

Co-infection profiling in this study identified a predominant association between GHPV and Goose circovirus (GoCV). This finding warrants significant clinical attention (22), as GoCV is a well-characterized immunosuppressive agent in waterfowl (23). The remarkably high co-detection rate of these two viruses suggests a potential pathogenic interplay in which GHPV may exacerbate the clinical sequelae of GoCV infection. Whether a bona fide synergistic relationship exists between these two DNA viruses remains to be determined through controlled experimental challenge studies. Given the current lack of licensed vaccines and specific antiviral interventions, an integrated biosecurity strategy targeting multiple co-circulating pathogens appears to be a pragmatic approach for mitigating the disease burden in commercial flocks.

Genomic sequence analysis further demonstrated that GHPV possesses a high level of genetic conservation, with no evidence of prominent mutations, divergent evolutionary clades, or recombination events. These metrics of polymorphism analysis indicate a high level of genetic heterogeneity and a complex evolutionary structure within the global GHPV population. The VP1 gene, encoding the major capsid protein, was particularly stable at both the nucleotide-sequence and codon-usage levels. No strain-specific molecular signatures were identified among VP1 sequences. This high ENC value (> 40) suggests that the synonymous codon usage bias in the GHPV VP1 gene is relatively weak, implying that the viral translational efficiency may be governed more by mutational pressure or host-specific constraints than by intense selection for specific codons. These observations collectively support the conclusion that GHPV exhibits limited genetic variability and strong evolutionary conservation.

The epidemiological profile of GHPV in China is characterized by a low overall detection rate at the individual level, yet a high frequency of mixed infections, particularly with GoCV, alongside strong genomic conservation across strains. Several factors may contribute to this pattern. First, GHPV may possess relatively limited intrinsic pathogenicity and function primarily as an opportunistic or conditionally pathogenic agent, with disease expression amplified in the presence of co-infecting pathogens, as observed in adult geese with multiple concurrent infections. Second, its transmission dynamics or ecological niche may be closely associated with environments where mixed infections are prevalent, consistent with the substantial inter-farm variability detected in our nationwide survey. Third, the observed genomic stability may reflect purifying selection pressure; reliance on co-infection could reduce the evolutionary pressure acting on the viral genome, thereby favoring sequence conservation. Taken together, these observations suggest that the clinical impact of GHPV is mediated more by synergistic interactions with co-infecting agents than by high transmission frequency, providing a useful framework for future studies on viral ecology and pathogenesis.

Experimental findings further indicated that, among the tested goose embryos and corresponding primary cell cultures, only those derived from Landes geese were permissive for successful viral isolation. This observation suggests the possibility of strain-specific host–virus interactions. The successful isolation of this strain provides a critical resource for subsequent studies of GHPV pathogenicity and evolutionary biology and, to our knowledge, offers the first etiological evidence supporting the presence of this disease in China. This study has several limitations. The sample size may constrain the accuracy of prevalence estimates, and the use of conventional PCR rather than more sensitive quantitative PCR assays may have led to underestimation of viral positivity and precluded precise quantification of pathogen loads in mixed infections. In addition, controlled animal experiments are required to further elucidate the independent and synergistic pathogenic mechanisms of GHPV. Future research should continue to investigate the age-dependent pathogenicity and the molecular interactions between GHPV and GoCV, as well as the role of regional and seasonal factors in shaping outbreak patterns, to inform improved surveillance and hatchery management strategies.

## 5 Conclusion

In summary, this study provides a comprehensive characterization of a GHPV outbreak, establishing a definitive etiological link between GHPV infection and impaired hatchability in commercial waterfowl. To our knowledge, this is the first systematic investigation to delineate the epidemiological landscape of GHPV across major goose-producing regions in China. Our findings highlight a unique ecological pattern characterized by low environmental prevalence but a high frequency of synergistic co-infections, alongside remarkable genomic stasis. Furthermore, the identification of host-specific viral permissiveness in Landes geese provides a critical foundation for future mechanistic studies. Collectively, these results imply that GHPV is an emerging pathogen in the Chinese waterfowl industry, whose clinical impact is likely modulated through complex polymicrobial interactions. These insights underscore the urgent need for sustained molecular surveillance in both geese and ducks and the strain-matched vaccines to mitigate the substantial economic risks posed by GHPV.

## Data Availability

The original contributions presented in the study are publicly available. This data can be found here: the sequence data generated in this study has been deposited in GenBank under the accession number PX932420.
