# Supplementary material for: Molecular epidemiology and characterization of goose polyomavirus in China: insights into its impact on hatchability and susceptibility to co-infections
Source: Front Vet Sci. 2026 May 12;13:1810451. doi: 10.3389/fvets.2026.1810451 (PMC13203973; doi:10.3389/fvets.2026.1810451)
Supplement: Supplementary file 1 [file Supplementary_file_1.zip › MSID1810451_Supplementary_Material/Supplementary Table 1.docx]

| **Sampling**  **years** | **Province** | **Detection Date** | **Farm** | **Sample  type** | **GHPV detection Result** | **Number of Positive Samples** | **Positive Rate** | **Number of Samples** | **Other pathogens detected** |
| --- | --- | --- | --- | --- | --- | --- | --- | --- | --- |
| 2023 | Sichuan | 2023.3 | Hatchery | unhatched goose embryos | Positive | 30 | 19 | 63% | - |
| 2023 | Jiangsu | 2023.4 | breeder goose farm | goslings | Positive | 30 | 2 | 7% | - |
| 2023 | Shandong | 2023.5 | meat-type goose farm | goslings | Positive | 60 | 1 | 2% | - |
| 2023 | Anhui | 2023.8 | breeder goose farm | goslings | Positive | 30 | 1 | 3% | - |
| 2023 | Jiangsu | 2023.9 | meat-type goose farm | dead adult geese | Positive | 1 | 1 | 100% | GHPV(+)、ASTV(+) |
| 2023 | Jiangsu | 2023.9 | meat-type goose farm | dead adult geese | Positive | 1 | 1 | 100% | - |
| 2023 | Yunnan | 2023.9 | meat-type goose farm | dead adult geese | Positive | 1 | 1 | 100% | GPV(+)、GHPV(+)、GOCV(+) |
| 2023 | Shandong | 2023.11 | breeder goose farm | goslings | Positive | 80 | 3 | 4% | - |
| 2023 | Shandong | 2023.11 | breeder goose farm | goslings | Positive | 50 | 2 | 4% | - |
| 2024 | Sichuan | 2024.1 | breeder goose farm | dead adult geese | Positive | 5 | 5 | 100% | GOCV(+)、ASTV(+)、GHPV(+) |
| 2024 | Yunnan | 2024.2 | breeder goose farm | goslings | Positive | 25 | 2 | 8% | - |
| 2024 | Yunnan | 2024.2 | breeder goose farm | goslings | Positive | 25 | 2 | 8% | - |
| 2024 | Sichuan | 2024.3 | breeder goose farm | dead adult geese | Positive | 1 | 1 | 100% | FAV(+)、GHPV(+)、ASTV(+)、GOCV(+)、NDPV(+) |
| 2024 | Sichuan | 2024.3 | breeder goose farm | dead adult geese | Positive | 1 | 1 | 100% | FAV(+)、GHPV(+)、ASTV(+)、GOCV(+)、NDPV(+) |
| 2024 | Fujian | 2024.3 | breeder goose farm | dead adult geese | Positive | 30 | 1 | 3% | - |
| 2024 | Fujian | 2024.3 | meat-type goose farm | dead adult geese | Positive | 1 | 1 | 100% | FAV(+)、GHPV(+)、ASTV(+)、GOCV(+)、NDPV(+) |
| 2024 | Fujian | 2024.3 | meat-type goose farm | dead adult geese | Positive | 1 | 1 | 100% | FAV(+)、GHPV(+)、ASTV(+)、GOCV(+)、NDPV(+) |
| 2024 | Sichuan | 2024.5 | meat-type goose farm | goslings | Positive | 29 | 2 | 7% | - |
| 2024 | Sichuan | 2024.5 | meat-type goose farm | goslings | Positive | 14 | 1 | 7% | - |
| 2024 | Anhui | 2024.6 | meat-type goose farm | goslings | Positive | 35 | 3 | 9% | - |
| 2024 | Anhui | 2024.6 | meat-type goose farm | goslings | Positive | 22 | 2 | 9% | - |
| 2024 | Anhui | 2024.6 | meat-type goose farm | goslings | Positive | 11 | 1 | 9% | - |
| 2024 | Yunnan | 2024.7 | meat-type goose farm | dead adult geese | Positive | 1 | 1 | 100% | GOCV(+)、ASTV(+)、GHPV(+) |
| 2024 | Yunnan | 2024.7 | meat-type goose farm | dead adult geese | Positive | 1 | 1 | 100% | GOCV(+)、ASTV(+)、GHPV(+) |
| 2024 | Yunnan | 2024.7 | meat-type goose farm | dead adult geese | Positive | 1 | 1 | 100% | NDRV(+)、GOCV(+)、ASTV(+)、GHPV(+) |
| 2024 | Yunnan | 2024.8 | meat-type goose farm | dead adult geese | Positive | 1 | 1 | 100% | GHPV(+)、GOCV(+)、ASTV(+) |
| 2024 | Sichuan | 2024.8 | meat-type goose farm | goslings | Positive | 38 | 3 | 8% | - |
| 2024 | Sichuan | 2024.8 | meat-type goose farm | goslings | Positive | 25 | 2 | 8% | - |
| 2024 | Sichuan | 2024.8 | meat-type goose farm | goslings | Positive | 13 | 1 | 8% | - |
| 2024 | Fujian | 2024.9 | meat-type goose farm | dead adult geese | Positive | 80 | 2 | 3% | - |
| 2024 | Shandong | 2024.9 | meat-type goose farm | dead adult geese | Positive | 100 | 5 | 5% | - |
| 2024 | Shandong | 2024.9 | meat-type goose farm | dead adult geese | Positive | 80 | 4 | 5% | GHPV（+）、GOCV（+） |
| 2024 | Yunnan | 2024.11 | meat-type goose farm | dead adult geese | Positive | 1 | 1 | 100% | GOCV(+)、GHPV(+) |
| 2024 | Yunnan | 2024.11 | meat-type goose farm | dead adult geese | Positive | 1 | 1 | 100% | GOCV(+)、NDPV(+)、GHPV(+) |
| 2024 | Shandong | 2024.11 | meat-type goose farm | dead adult geese | Positive | 1 | 1 | 100% | GHPV(+)、GOCV(+) |
| 2025 | Yunnan | 2025.2 | Hatchery | unhatched goose embryos | Positive | 30 | 1 | 3% | - |
| 2025 | Shandong | 2025.2 | meat-type goose farm | dead adult geese | Positive | 1 | 1 | 100% | GHPV(+)、ASTV(+) |
| 2025 | Shandong | 2025.3 | meat-type goose farm | dead adult geese | Positive | 1 | 1 | 100% | GPV(+)、GHPV(+)、GOCV(+) |
| 2025 | Anhui | 2025.3 | meat-type goose farm | dead adult geese | Positive | 1 | 1 | 100% | GPV(+)、GHPV(+)、GOCV(+) |
| 2025 | Anhui | 2025.3 | meat-type goose farm | dead adult geese | Positive | 1 | 1 | 100% | GHPV(+)、GOCV(+) |
| 2025 | Shandong | 2025.4 | meat-type goose farm | dead adult geese | Positive | 1 | 1 | 100% | NDPV(+)、GOCV(+)、GHPV(+) |
