# Supplementary material for: Molecular epidemiology and characterization of goose polyomavirus in China: insights into its impact on hatchability and susceptibility to co-infections
Source: Front Vet Sci. 2026 May 12;13:1810451. doi: 10.3389/fvets.2026.1810451 (PMC13203973; doi:10.3389/fvets.2026.1810451)
Supplement: Supplementary file 1 [file Supplementary_file_1.zip › MSID1810451_Supplementary_Material/Supplementary Table 3, 4, 7.DOCX]

Table S2 Overall prevalence of co-infecting pathogens

| **Co-infecting Agent** | **Cases ( n)** | **Prevalence (%)** |
| --- | --- | --- |
| Goose Circovirus (GoCV) | 25 | 93% |
| Goose Astrovirus (GoAstV) | 15 | 56% |
| Newcastle Disease Virus (NDV) | 6 | 22% |
| Fowl Adenovirus (FAdV) | 4 | 15% |
| Goose Parvovirus (GPV) | 3 | 11% |
| Novel Duck Reovirus (NDRV) | 1 | 4% |

Table S3 Specific co-infection combinations

| Infection Type | Cases (n) | Proportion of Mixed Infections (%) | Pathogen Combination | Cases within Subtype (n) | Proportion within Subtype (%) |
| --- | --- | --- | --- | --- | --- |
| Dual infection | 9 | 33% | GHPV + GoCV | 7 | 78% |
|  |  |  | GHPV + GoAstV | 2 | 22% |
| Triple infection | 13 | 48% | GHPV + GoAstV + GoCV | 8 | 62% |
|  |  |  | GHPV + GPV + GoCV | 3 | 23% |
|  |  |  | GHPV + GoCV + NDV | 2 | 15% |
| Quadruple infection | 5 | 19% | GHPV + FAdV + GoAstV + GoCV | 4 | 80% |
|  |  |  | GHPV + NDRV + GoCV + GoAstV | 1 | 20% |

Table S7 Polymorphism Indicators

| Polymorphism Indicators | Specific Values |
| --- | --- |
| Average Nucleotide Difference (*K*) | 11.148 |
| Overall Nucleotide Diversity (*π*) | 0.00212 |
| Number of Haplotypes (*h*) | 31 |
| Haplotype Diversity (*Hd*) | 0.995±0.009 |
| Variance of Haplotype Diversity | 0.00007 |
